# Low energy expenditure and resting behaviour of humpback whale mother-calf pairs highlights conservation importance of sheltered breeding areas

**DOI:** 10.1038/s41598-018-36870-7

**Published:** 2019-01-25

**Authors:** L. Bejder, S. Videsen, L. Hermannsen, M. Simon, D. Hanf, P. T. Madsen

**Affiliations:** 1 Cetacean Research Unit, School of Veterinary and Life Sciences, Murdoch University, Murdoch, WA Australia; 2 Marine Mammal Research Program, Hawaii Institute of Marine Biology, University of Hawaii at Manoa, Kaneohe, Hawaii United States; 3 Centre for Sustainable Aquatic Ecosystems, Harry Butler Institute, Murdoch University, Murdoch, WA Australia; 4 Zoophysiology, Department of Bioscience, Faculty of Science and Technology, Aarhus University, Aarhus, Denmark; 5 Greenland Climate Research Centre, Greenland Institute of Natural Resources, Nuuk, Greenland; 6 Aarhus Institute of Advanced Studies, Høegh-Guldbergs Gade 6B, 8000 Aarhus, Denmark

## Abstract

Understanding the behaviour of humpback whale mother-calf pairs and the acoustic environment on their breeding grounds is fundamental to assessing the biological and ecological requirements needed to ensure a successful migration and survival of calves. Therefore, on a breeding/resting ground, Exmouth Gulf, Western Australia, we used animal-borne DTAGs to quantify the fine-scale behaviour and energetic expenditure of humpback whale mothers and calves, while sound recorders measured the acoustic environment. We show that: (i) lactating humpback whales keep their energy expenditure low by devoting a significant amount of time to rest, and their use of energy, inferred from respiration rates, is ~half than that of adults on their foraging grounds; (ii) lactating females mainly rest while stationary at shallow depths within reach of the hull of commercial ships, thus increasing the potential for ship strike collisions; (iii) the soundscape is dominated by biological sources; and (iv) even moderate increases of noise from vessels will decrease the communication range of humpback whales. Planned commercial infrastructure in Exmouth Gulf will cause a substantial increase in shipping traffic with the risk of ship strikes and acoustic disturbance potentially compromising energy reserves for the southern migration of humpback whales.

## Introduction

Individual and population fitness is partly predicated on a balance between energy intake and expenditure, energy transfer to offspring, and predation mitigation^1^. Wildlife can be categorized as income or capital breeders based on their life history strategies for energy intake and expenditure, and for allocation of resources into their long-term reproduction and survival^2,3^. Income breeders replenish their energy reserves concurrently with reproduction, whereas capital breeders finance the energy cost of reproduction through stored energy reserves^3^. Most mysticetes (baleen whales) are capital breeders that typically breed in low-productive lower latitudes and feed in highly-productive higher latitudes. While geographic and temporal scales of animal migrations vary considerably, all are functional adaptations to spatio-temporal fluctuations in resource availability (e.g., prey, mates and optimal habitat for successful reproduction) and predation pressures^4^.

For baleen whales, these trade-offs have evolved into extreme migrations between spatially and temporally decoupled breeding and foraging areas^5^. Humpback whales (*Megaptera novaeanglea*) carry out some of the longest migrations on earth. For example, the southern hemisphere humpback whale Breeding Stock D migrates annually approximately 8,500 km from their breeding grounds in north-west of Western Australia (WA) to their feeding grounds in the Antarctic Management Area IV^6,7^. In the 1960s, this stock was decimated to critically low numbers (<300 individuals) during the modern whaling era^8^. Since the moratorium of commercial whaling in 1982, the stock has recovered significantly, with estimated annual population increases of 8–12% between 2008 and 2012^9,10^. In 2008, estimates of the population size ranged between 19,200–33,850 individuals^9–11^. The population recovery is hailed as a conservation success, and provides an example of management interventions that result in a positive outcome, creating hope and contributing to ‘ocean optimism’^12^. It has been suggested that available undisturbed breeding/resting habitat along the WA coastline may partly explain the high population growth rate measured in WA’s Stock D^13^, but little data exist to qualify this critical notion.

**Figure 1:**
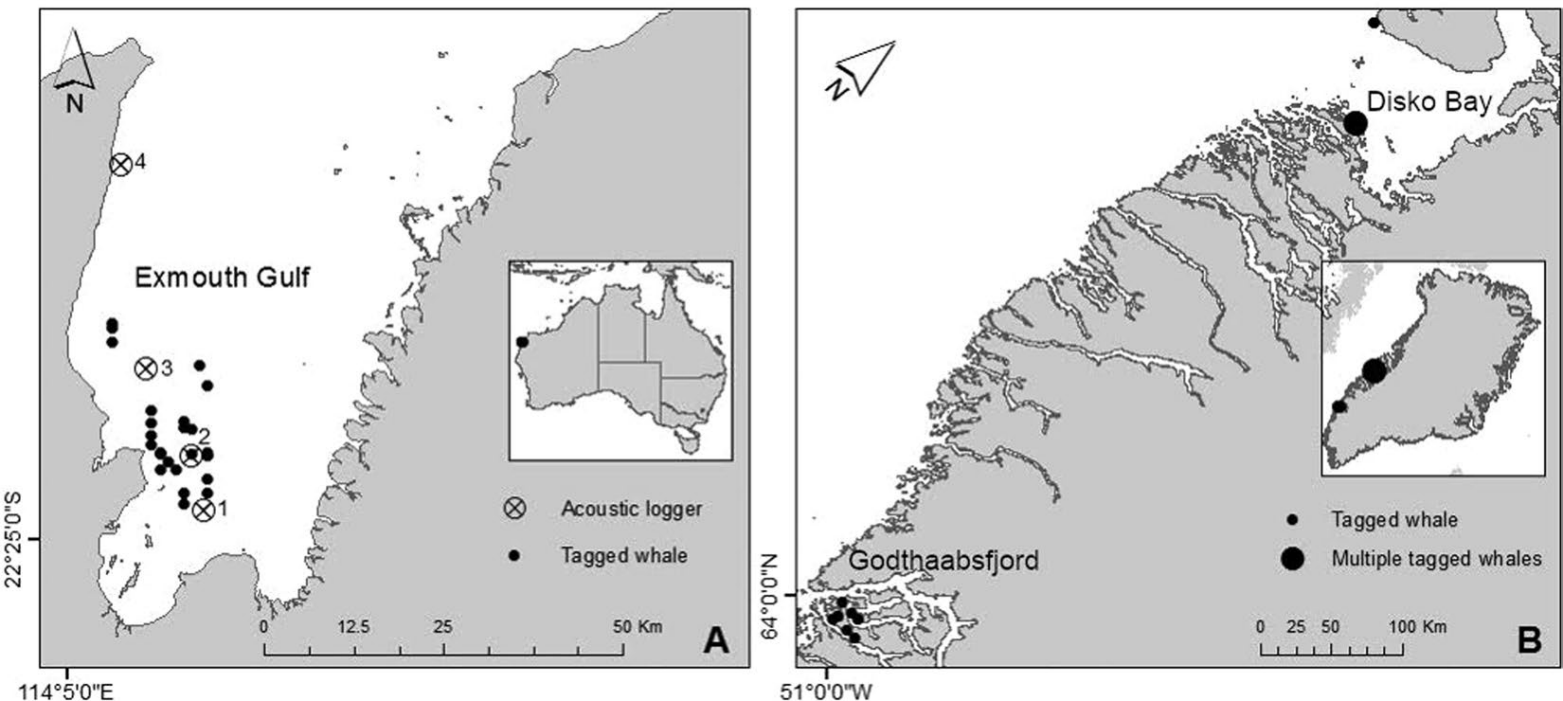
Study sites. (**A**) Exmouth Gulf, Western Australia: a known humpback whale breeding/resting area. (**B**) Godthaabsfjord and Disko Bay, Greenland part of the West Greenland humpback whale feeding ground. The location of whale tagging events and acoustics logger deployments in Exmouth Gulf (Stations 1–4) are depicted.

Exmouth Gulf (Fig. 1) and environs on the WA coastline serves as a resting and breeding area likely needed for Stock D humpback whale mothers to nurse their calves to enable them to gain adequate energy reserves before their annual southern migration^6,7,14^. Specifically, Exmouth Gulf provides calm waters and protection from predators and from open oceanographic conditions during the prevailing south-easterly winds between August and November – coinciding with the peak number of whales in the Gulf during the southern migration^6,13^.

However, Exmouth also serves as an important onshore location to support and accommodate the hydrocarbon extraction industry, which exploit large-scale deposits located to the north of the Gulf (i.e., the north-west shelf). Current development plans in the Gulf include a multi-purpose deep water wharf, cruise ship tourism, export of limestone and agriculture products, and to expand the capabilities of the defence industry^15^. Such expansions will see a substantial increase in marine traffic and a concomitant increase in anthropogenic noise within humpback whale breeding/resting habitat, with the potential for increased risk of ship strikes and acoustic disturbance to resting and nursing mother and calf whales. Noise levels in marine environments worldwide have increased considerably in the past decades as a result of increased anthropogenic marine activities^16,17^. Noise pollution, largely produced by the shipping industry, is considered a major contributor to habitat degradation in the marine environment^18,19^.

Detailed insights into the behaviour of humpback whale mothers and calves and the acoustic environment on breeding/resting grounds are important to better understand their biological and ecological requirements; and to inform management in a region slated for human activities that have potential adverse effects on whales within important habitats. Here, within a humpback whale breeding/resting area, we aimed to quantify (i) fine-scale undisturbed behaviour to better inform management about biological and ecological requirements of humpback whales; (ii): energetic expenditure to investigate how mothers and calves optimally prepare for a long migration, and (iii) current ambient noise levels to investigate how introduction of anthropogenic noise sources can potentially affect vocal communication between mothers and calves to ensure close proximity, and between adult males and females to communicate mating information. For the sake of comparison, (i) and (ii) were also quantified on a known humpback whale foraging ground.

## Results

A total of 42 humpback whales were tagged; 25 whales on their breeding/resting habitat in Exmouth Gulf (n = eight neonate calves, 16 lactating females and one adult male; Table S1 and Fig. S1) and 17 adults on foraging grounds in Godthaabsfjord and Disko Bay, Greenland (Table S1 and Fig. S2). All whales were tagged between the blowhole and dorsal fin, except for three that were tagged behind the dorsal fin. Tags stayed on whales for a mean (±s.d.) of 11.9 ± 8.6 hrs and 10.0 ± 7.5 hrs, in Exmouth and Greenland, respectively.

### Dive behaviour

#### Behavioural activity on breeding/resting and foraging ground

**Figure 2:**
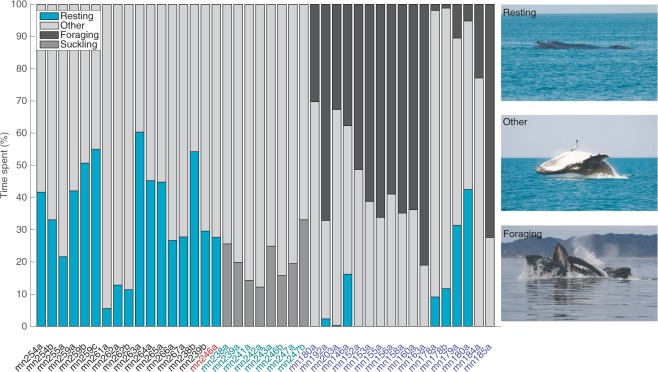
Behavioural budget of 25 whales: 16 lactating females (black); one adult male (red); and eight neonate calves (light green) on the Exmouth breeding/resting ground; and 17 adult whales (blue) on the West Greenland foraging ground. Behaviour was inferred based on DTAG accelerometers, focal follows and Minimum Specific Acceleration (MSA).

The behaviour (resting, foraging, other or suckling) of whales on their breeding/resting ground was inferred based on DTAG accelerometers, Minimum Specific Acceleration (MSA; sensu^20^; Methods Section). Tagged whales returned to resting/logging behaviour within 11 min (median) of being tagged (quantified via the MSA signatures; See Materials and Methods). On the breeding ground, lactating females (n = 16) spent on average 35% of time resting (range: 6–60%) and 65% of time “other” (range: 40–94%) (Fig. 2). Neonate calves spent on average 21% of time suckling (range 12–33%)^21^ and 79% of time “other” (range 67–88%). The one adult male spent 28% of time resting and 72% of time “other”. Adult whales were never observed foraging on their breeding grounds. On the foraging grounds, adults spent on average 42% of time foraging (range 1–81%), 6% of time resting (range 0–41%) and 52% of time “other” (range 19–98%) (Fig. 2).

#### Dive profiles from breeding/resting and foraging grounds

**Figure 3:**
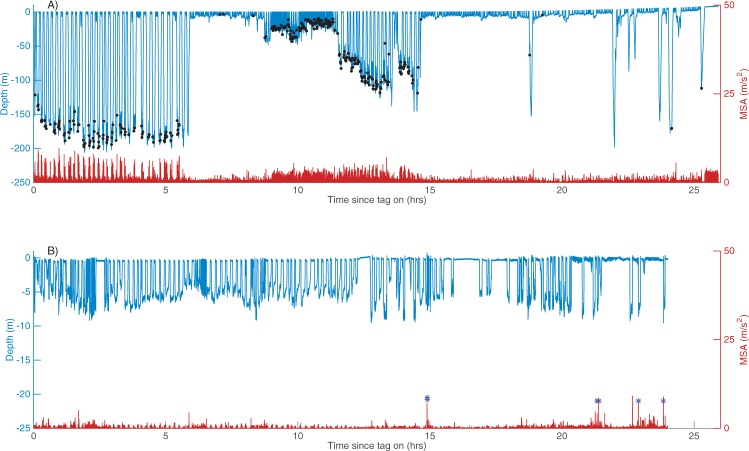
Typical dive profile of (**A**) a foraging humpback whale (ID: mn07_203a; Greenland), foraging lunges indicated by black dots, and (**B**) a resting lactating female (ID: mn13_265a) in Exmouth Gulf (**B**). Note lack of movement at depth as indicated by the MSA (in red); surface active behaviour (e.g. breach and tail slaps) indicated by blue stars.

Dive behaviour differed between the breeding/resting and foraging grounds (Figs 3 and S1, S2), partly due the behaviour of the animals in each habitat (Fig. 2). Whales exerted more energy (as inferred from the MSA readings) on the foraging ground compared to the breeding grounds (Figs 3 and S1, S2).

**Figure 4:**
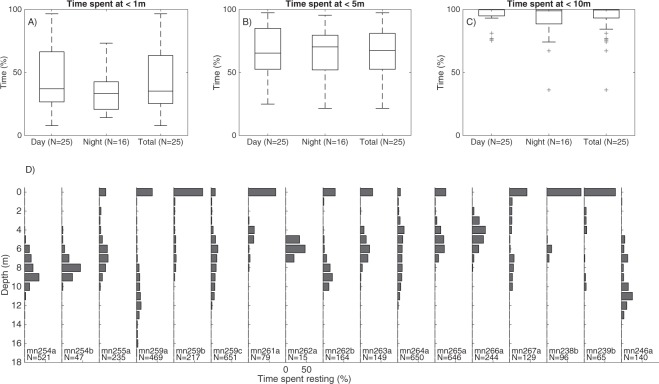
(**A**–**C**) Percentage of time that humpback whales spent at <1 m, <5 m and <10 m during night and daytime for all whales (16 lactating females, one adult male, 8 neonate calves) on the breeding/resting ground (n = 25), all behavioural categories included, N indicates the number of whales included in the analysis, in the Total category depths from both day and night periods are included. (**D**) Depth at which adult humpback whales rest on breeding/resting grounds (n = 16 lactating females, n = 1 adult male), N indicates amount of minutes spent resting.

Dive profiles of lactating females, their neonate calves and one adult male on a breeding/resting ground showed individual variation, with no strong diurnal dive patterns (Figs S1 and 4A–C)^21^. Sample sizes of tagged whales at night were low on their foraging grounds, and thus we are not able to make inferences on diurnal dive patterns (Fig. S2). On the breeding ground, lactating females spent significantly (two-sample t-test, *F*_1,15_ = 1.306, p = 0.0207) more time (53%; SD = 18) within 3 m of the surface, across all behavioral categories, than whales on their feeding grounds (39%; SD = 16). Of the average 35% of time spent resting by lactating females in Exmouth Gulf (Fig. 2), they spent the majority (>75%) of resting time below the surface, mostly at 4–9 m depth (Figs 4D and S1).

### Respiration rates

**Figure 5:**
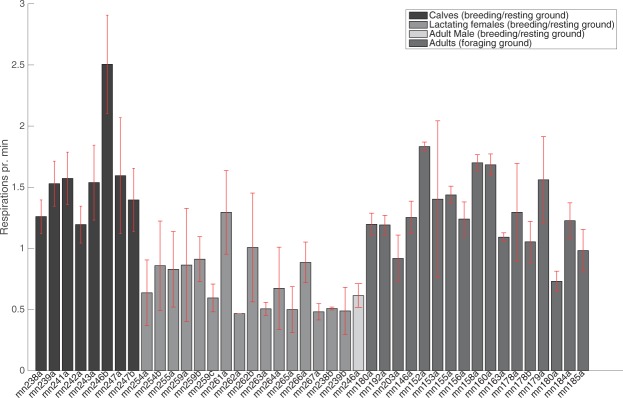
Respiration rates (respirations per min) of 25 whales (8 neonates, 16 lactating females, 1 adult male) on a breeding/resting ground in Exmouth, WA, and 17 adult whales on foraging grounds in Godthaabsfjord and Disko Bay, West Greenland. Red lines indicate standard deviation.

Respiration rates of lactating females (mean (±s.d.) (0.7 ± 0.2 respirations/min) on the breeding/resting ground were significantly lower (two-sample t-test, *F*_1,15_ = 0.664, p = 1.2775e-06) than adult whales on their foraging grounds (1.3 ± 0.3 respirations/min) (Fig. 5). On the breeding/resting ground, the respiration rate of neonate calves (1.6 ± 0.4 respirations/min) were significantly higher than their lactating mothers (two-sample t-test, *F*_1,15_ = 0.354, p = 1.46e-06).

#### Soundscape

**Figure 6:**
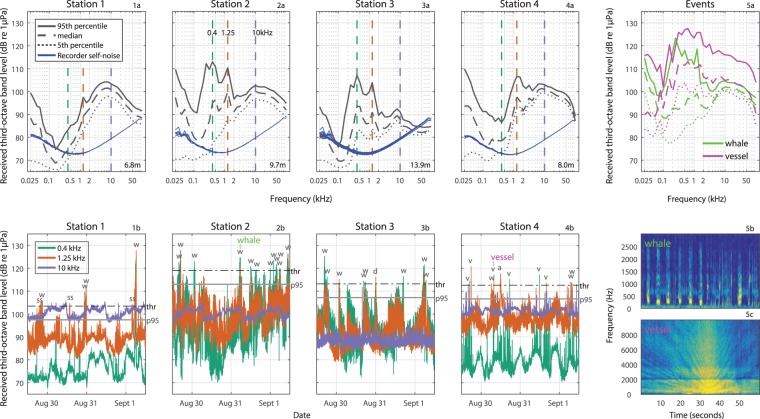
Ambient noise continuously recorded at four recording stations (station 1–4) for three days on Exmouth resting/breeding ground, quantified as third-octave sound pressure levels (TOLs) in dB re 1 μPa RMS (1-minute integration window). Water depth at each station is indicated in right bottom corners on upper panel plots. Upper panel (1–4a): recorded TOLs are shown as percentiles (5^th^, median and 95^th^) across 37 third-octave band frequencies (25 Hz–100 kHz). Self-noise of sound recorders is also plotted. Lower panel (1–4b): three third-octave bands (0.4, 1.25 and 10 kHz) with high ambient noise levels are shown in the time domain. Peak events above the threshold (the 95^th^ percentile (p95) +6 dB) at each station have been audited and marked according to the dominating sound source; ‘w’ = humpback whale, ‘d’ = delphinid, ‘v’ = vessel, ‘ss’ = snapping shrimp, ‘a’ = other anthropogenic noise. Two events (‘whale’ and ‘vessel’) are also shown as spectrograms (5b,c) and were further analysed into percentiles (5a; 1-second integration window).

All four recording stations had peaks in ambient noise levels around 1.25 kHz (Fig. 6, upper panels 1–4a, median: 89–100 dB re 1 µPa RMS, 95^th^ percentile: 97–110 dB re 1 µPa, RMS), which in most cases were dominated by humpback whale sounds, confirmed by auditing of recordings (Fig. 6, lower panels 1–4b). Humpback whales were the largest contributors to the peak in noise around 0.4 kHz at two stations (Station 2 and 3, Fig. 6, upper panels 2a and 3a, median: 94–95 dB re 1 µPa RMS, 95^th^ percentile: 107–113 dB re 1 µPa RMS) (Fig. 6, lower panels, 2b and 3b). Analysis of an event with clear humpback whale sounds further confirms that this species creates noise peaks in the third-octave bands centred around 0.4 and 1.25 kHz, and in the frequency band around 0.25 kHz (Fig. 6,5a). At Stations 2 and 3, noise around 0.25 kHz also reached high levels caused by humpback whales, although only temporarily (Fig. 6,2a, 95^th^ percentile: 112 dB re 1 µPa, Fig. 6,3a, 95^th^ percentile: 103 dB re 1 µPa), and not evident from median noise levels. At the station closest to Exmouth marina, Station 4, peaks in noise levels around 0.4 and 1.25 kHz were also recorded (Fig. 6,4b). These peaks were mainly caused by passing vessels or other anthropogenic activities. Analysis of a vessel event showed that vessels can cause a considerable broadband noise emission, including noise in third-octave bands around 0.25, 0.4 and 1.25 kHz overlapping with humpback whale sounds (Fig. 6,5a). Snapping shrimp contributed considerable noise (85–105 dB re 1 µPa RMS) to all stations in third-octave bands around 8–10 kHz throughout the recording period with daily fluctuations of 4–8 dBs, although with recorded noise levels being consistently approx. 10 dB lower at station 3. In summary, the soundscape in Exmouth Gulf was mainly dominated by biological sounds from wave action, humpback whales and snapping shrimp, with low noise contribution from shipping, boating and other anthropogenic activities.

## Discussion

This study used DTAGs to quantify the fine scale behaviour and energetic expenditure of humpback whale mothers and calves, and passive acoustic monitoring to quantify the acoustic environment on a whale breeding/resting ground planned for human development. We suggest that a potential increase in moving vessels and a concomitant increase in anthropogenic noise in Exmouth Gulf, will likely cause an increased risk of ship strikes and acoustic masking to resting and nursing mother and calf humpback whales. This may potentially compromise energy reserves for the upcoming migration and predator avoidance, with possible deleterious fitness consequences.

A recent study used multi-sensor DTAGs to quantify fine-scale neonate humpback whale suckling behaviour in Exmouth Gulf and documented that calf suckling is performed during 20.7 ± 7% of the total tagging time during which the mothers either rest at the surface or at depth^21^. Another recent study, also in Exmouth Gulf, assessed the body condition of lactating humpback whales from aerial photographs obtained from a non-invasive unmanned aerial vehicle (UAV)^22^. Photogrammetry methods were used to measure the surface area of individual whales, which was used as an index for body condition. The study documented a linear decline in the body condition of lactating females within the gulf. The significant decline in lactating female’s body condition implies substantial energetic costs of nursing a calf while simultaneously maintaining its own life functions. Here we show that lactating humpback whales accompanied by their suckling calves (<3 months of age) spend a significant amount of time resting on their breeding grounds. In line with that, we find that lactating humpback whales on the breeding/resting ground have about half the respiration rate, and therefore metabolic rate, compared to adult humpback whales on their foraging ground. Such low energy expenditure of humpback whales in Exmouth Gulf is likely critical to minimize the rate of decline in body condition of lactating females and optimizing calf growth to ensure a successful migration to their feeding ground. We suggest that this hypo-metabolic behaviour far away from feeding grounds may be compromised if the animals are disturbed above some level by elevated anthropogenic noise levels, shipping, boating and whale watching activities. This may thereby negatively influence energy transfer to the calves and their ability to successfully migrate back to feeding grounds. It follows that breeding areas provide important resting habitats needed for hypo-metabolic mothers to nurse their calves, to enable them to gain adequate energy reserves to continue their southern migration.

Over the past three decades, the global shipping industry has sustained continued expansion and growth in numbers, sizes and geographic routes^23^. At the same time, several whale populations have increased in size and range (e.g.^12,24^) resulting in a temporal and spatial overlap between whales and vessels. In turn, this has resulted in an apparent increase in vessel strikes of whales^25–29^. Accordingly, vessel strikes are one of the major conservation concerns to whale populations globally. Recent modelling research exemplifies the magnitude of the problem by showing that current levels of ship strike mortality of humpback whales, blue whales (*Balaenoptera musculus*) and fin whales (*Balaenoptera physalus*) off the U.S west coast are impeding population recoveries and causing population level effects^25^.

Approximately 15% of all vessel strikes to whales recorded globally have occurred in Australian waters, of which 59% involved humpback whales^29^. Our results show that lactating humpback whales (and their calves) in Exmouth Gulf spend considerable time resting (on average 35% of time) and stationary at depths within the reach of ship hulls. Thus out of sight from human observers, rendering the detection of whales nearly impossible which, in turn, increases the risk of potential collisions. Experimental work has provided clear evidence that some whales (e.g. North Atlantic right whales (*Eubalaena glacialis*)^30^) ignore approaching ships, increasing the risk of ship strike fatalities. Although this is a recovering population, concerns for their protection remain because anthropogenic activities in northern WA will only continue to increase. For instance, ship strikes are a major contributor to the lack of population recovery for the endangered North Atlantic right whale population^31^, and are exceeding the annual Potential Biological Removal (PBR) limits set by US National Marine Fisheries Service for three species of baleen whales off California^25^, including humpback whales.

The soundscape in Exmouth Gulf is mainly dominated by biological sounds from snapping shrimp and humpback whales, with very little noise contribution from anthropogenic sources, including vessels (Fig. 6). Humpback whale sounds mainly contribute ambient noise in third-octave bands around 0.4 and 1.25 kHz. They also contribute considerable noise in the third-octave band around 0.25 kHz (Fig. 6,5a), although only within shorter ranges of the vocalizing whales. This is due to the shallow water depths in this environment (7–14 m) that gives rise to a high-pass filter effect with a low frequency cut-off below 100–200 Hz^32^. Noise below 100 Hz was created locally around each recorder by wind and waves^19,33^. Snapping shrimp dominated noise in third-octave bands around 8–10 kHz at all four stations, although with 10 dB lower noise levels at station 3, which may result from the larger water depth or other environmental conditions that affect snapping shrimp density^34^ at this station.

The few vessels passing each recorder during the recording period resulted in considerable elevations in ambient noise levels across a broad range of frequencies, including third-octave bands overlapping with humpback whale sounds (0.25, 0.4 and 1.25 kHz; Fig. 6,5a,c). These results highlight that noise from vessels has the spectral overlap and levels to cause acoustic masking of humpback whale communication sounds in Exmouth Gulf. Acoustic masking effects can be estimated by using the range reduction factor^35^. This approach uses the passive sonar equation, where the ability of an animal to detect a sound signal in noise depends on a certain signal-to-noise ratio (SNR), which is determined by the source level of a signal (SL), the transmission loss (TL), the noise level (NL) and the auditory properties of the receiver (RP): SNR = SL − TL − NL + RP. If SL and RP can be assumed to be independent and constant for communication events, an animal exposed to increased noise (NL) will face a reduction in active space^36^ by the same amount on a dB scale. For example, an elevation in noise of 10 dB will, all other things equal, reduce SNR and thereby detection range (the so-called *active space*;) by 10 dB corresponding to a drop of 70%, if assuming spherical spreading, whereas the area over which a signal can be detected or decoded will drop by an order of magnitude (i.e. 90%)^36,37^. This emphasizes that even moderate increases in ship noise in Exmouth Gulf can considerably decrease the communication ranges of humpback whales. Maintaining acoustic contact is important for the communication between adult females and males, whose mating strategy rely heavily on acoustics likely via sexual selection on song^38^ and for the critical communication between mothers and calves, where constant contact is crucial for the survival of the calf. In Exmouth Gulf, neonate humpback whale calves produce contact calls to their mothers of low source levels (mean of 141 ± 1 dB re 1 µPa RMS for tonal sounds and 136 ± 4 dB re 1 µPa RMS for grunting sounds) with an active space of less than 100 meters^21^. The low source levels are likely to optimize acoustic crypsis to reduce the chance of detection by predatory killer whales^39^ and male escort humpback whales that may disrupt suckling and resting opportunities^21^. Median noise levels in Exmouth Gulf in the third-octave band around 800 Hz, where calves use tonal sounds^21^, are approx. 92 dB re 1 µPa (RMS) (Fig. 6,2b, median), thus a 10 dB increase in noise would result in noise levels of 102 dB re 1 µPa (RMS) in that frequency band. Such an increase in noise can be caused by large ships within 1000–2000 meters range, depending on speed, propellers and other vessel characteristics^40^. Longer ranges to moving vessels will correspondingly make the range reduction factor smaller, but still have substantial effects on the active space. Compensatory mechanisms, such as call redundancy and increased vocal output levels driven by the Lombard response, may partly offset negative effects of masking from vessel noise, but evoking these mechanisms also comes at a cost. Noise propagation in this shallow water environment with varying water depth, bottom composition and sea states is complex over longer ranges. While it is difficult to make accurate predictions about noise loads given the complex interactions between vessel behavior and varying propagation conditions, it is safe to say that increased vessel traffic will increase masking in pertinent frequency bands at levels that are relevant to humpback whale active space. Future research will hopefully shed light on the degree to which exposed mother-calf pairs and singing males employ compensatory mechanisms to increases in masking noise.

Ship noise exposure may cause behavioural responses in humpback whales, which can affect individual fitness by disrupting vital behaviours, such as resting or feeding. Reported responses include alterations in dive behaviour^41–43^, changes in vocal behaviour^44,45^, and displacement^45,46^, even at exposure ranges of 3–4 km from a vessel^41^. Ship traffic has been found to have a larger impact on pods with calves than pods without calves^42^ and mother-calf pairs are considered the most sensitive individuals within a humpback whale population^47,48^. Their responses to vessel noise may include increased movement speeds and decreased respirations of mothers, less active behavioural events (e.g. rolling) in calves, and decreased time spent resting in both mother and calf^49^. There are no indications that resting mother-calf pairs habituate to vessel noise, and even less sensitive feeding adults have not habituated to shipping although exposed for decades^43^. Instead, close vessel approaches have been indicated to cause sensitization in calves, which thereby elicit even stronger behaviours in future exposures^42^. Vessel noise can thereby affect the energy budgets of mother-calf pairs both by masking crucial communication and/or by disturbing vital resting or nursing time. Even single vessel events causing a disruption in normal calf behaviour are expected to carry energetic costs^46^, and with repeated exposures to vessel noise, the energy transfer and predator avoidance behavior essential for successful migration of both mother and calf may be compromised.

The Western Australian humpback whale population is recovering from commercial whaling^12–19^. Such recovery may rely critically on the available undisturbed breeding/resting habitat of Exmouth Gulf where hundreds of mother-calf pairs visit annually^13^. The close proximity of Exmouth Gulf to the hydrocarbon fields of Australia’s north west shelf make the gulf an attractive location for support facilities to accommodate the extraction industry. There are current plans for the development of a multi-purpose deep water wharf within Exmouth Gulf to facilitate coastal processing facilities that service the oil and gas resource industries, cruise ship tourism, export of limestone and products from agriculture industries, and to expand the current Australian and United States defence industry, including the ability to anchor and service naval destroyer-size warships^15^. A causeway extending approximately 1 km into Exmouth Gulf is being proposed with two deep-water wharves to be built. In addition, whale watch tourism focusing on humpback whales is also expanding, including a recently completed “swim-with-humpback-whale” trial in the region^50^. While this trial and current whale watch activities occur mainly on the western side of the Exmouth Peninsula, there is increasing interest to expand the tourism industry to include the Exmouth Gulf whale breeding/resting grounds. Combined, such activities and new infrastructure will see a significant increase in shipping traffic and recreational vessel activity, and a concomitant increase in anthropogenic noise within humpback whale breeding/resting habitat in Exmouth Gulf. This will increase the risk of ship strikes and acoustic disturbance/masking effects to resting and nursing mother and calf humpback whales. These effects potentially compromise energy reserves crucial for their upcoming migration and may also decrease predator avoidance, and hence have serious fitness impacts.

There is strong evidence to suggest that altering/re-routing shipping lanes is an effective way to decrease the spatial and temporal co-occurrence of whales and ships which, in turn, reduces the risk of collisions^51,52^ and the potential for acoustic masking effects. However, modification to shipping routes is costly and not always feasible, but in the case of Exmouth Gulf it does seem prudent to explore how shipping lanes can overlap the least with preferred routes of migration and resting of humpback whales. In response, alternative measures have been implemented to reduce the risk of whale collisions, including vessel speed reductions^52–55^. A reduction in vessel speed both reduces whale encounter rates^56^ and the blunt impact forces involved in a collision^57^, which, in turn decreases the probability of serious injuries. In addition, a reduction in vessel speed also reduces ship noise levels, in particular if the speeds are reduced to a level where cavitation is avoided^58,59^. It is primarily frequencies above some 500 Hz that will propagate efficiently into the shallow areas preferred by the mother-calf pairs, meaning that slow moving vessels that produce little energy at high frequencies, when their propellers are not causing cavitation, will lead to much lower effective exposure levels compared to higher speeds at the same ranges. Slower speeds will therefore both reduce the risk of whale collisions and lead to a smaller range reduction factor and risk of behavioural disruption. While low levels of anthropogenic noise in the ocean is desirable, it also sets up a dichotomy between having ship noise at levels that cause minimal behavioural responses by whales and minimal masking of their vocal communication signals, while still being audible for animals at ranges giving them sufficient time to react and displace to avoid ship collisions. Unfortunately, there is a limited understanding of how resting, submerged humpback whales respond to approaching ships and associated noise. Therefore, given the importance of Exmouth Gulf to lactating humpback whales and their calves, future research should target this knowledge gap. Research findings from such studies will help inform on appropriate conservation measures aimed to decrease the risk of ship strike collisions and effects of noise pollution on humpback whale mothers and calves on an important breeding/resting ground.

## Materials and Methods

We deployed high resolution onboard multi-sensor DTAGs (Version 2 and 3)^60^ on humpback whales to quantify the behaviour and activity of humpback whale mom-calf pairs, including behavioural states, dive depths and durations, as well as metabolic energy expenditure (via respiration rates) on a breeding/resting ground (Exmouth Gulf, Western Australia; Fig. 1) and a feeding ground for comparison (Godthaabsfjord and Disko Bay, West Greenland^61^; Fig. 1). Specifically, we suction-cup tagged neonate calves, their mothers and one adult male on a breeding/resting ground, and adults on the foraging ground. We also deployed acoustic noise loggers to quantify the ambient soundscape in Exmouth Gulf during the peak season for mother and calf occurrence.

### Field sites and tagging procedures

#### Breeding/resting ground

Field work was conducted in Exmouth Gulf (~4,000 km^2^; mean and max depth of 9 m and 20 m, respectively), WA, (22.16°S, 114.30°E) (Fig. 1) during August and September 2013 and 2014. This study was conducted from a 5.5 m aluminium-hulled boat powered by an 80 HP Yamaha four-stroke engine. The research vessel was beach-launched from the south-western coastline of the gulf in order to access mother-calf pairs within its inner-most reaches, which minimized the risk of a tagged whale leaving the gulf and the tag detaching on Ningaloo Reef west of the Exmouth Peninsula. When a resting mother-calf pair was located, we performed an hour of behavioural focal follow on the pair prior to and after tagging in order to uncover possible effects of tagging and to ground-truth behaviour inferred from the inertial and pressure sensors of the tag. The focal follow was conducted at a minimum distance of 200 m using naked eye and binocular observations of surface behaviour and breathing, and observations were relayed verbally to the audio track of a head-mounted GoPro Hero 3 by an experienced observer.

When tagging, the resting mother-calf pair was approached slowly (<2 knots) from a rear aspect, until the boat was parallel to the mother’s dorsal fin at a distance of 6–8 m. The tag was then placed between the blowhole and the dorsal fin, using a 9 m hand-held carbon fiber pole. We used non-invasive, version 3 DTAGs with four 40 mm diameter, soft silicone suction cups. Tags detached either pre-maturely due to whales rubbing or after a pre-programmed period of 22 h using a timed burn wire release. The tags were retrieved the following day using radio tracking of the integrated 220 MHz VHF beacon in the tag; first from a 200 m high vantage point on land (yielding a 30 nm tracking range) and then subsequently from the research vessel (5 nm tracking range), when the overall bearing line from a known GPS point on land and the tag was established. The DTAGs sampled three-axes accelerometers, magnetometers and a pressure sensor at 200 Hz with 16 bit resolution, and stereo sound at 120 kHz, 16 bit resolution, rendering a flat (±2 dB) frequency response between 0.4 and 45 kHz.

#### Foraging ground

Field work was conducted in two locations on the West Greenlandic humpback whale feeding ground^62^; In Godthaabsfjord (64.2°N, 51.8°W) (Fig. 1), during July 2007, May/June 2008 and in Disko Bay (69.0°N, 52.0°W), in June/July 2012. Whales were approached slowly from aluminium vessels (<7 m) equipped with Yamaha 100–150 hp, 4 stroke outboard engines. DTAGs versions 2 and 3 were attached to whales with suction cups using a 7 m hand-held carbon fiber pole (in 2007) or a 12 m cantilevered carbon fiber pole (in 2008 & 2012)^20,63^. The Greenland humpback whale population is clearly not part of the Australian Stock D population and it is possible the two have developed stock-specific strategies and regional adaptations. Nevertheless, given the stereotypic foraging behaviour of humpback across areas and stocks, a comparison of the behavioural budgets of whales on foraging and breeding/resting grounds from different populations still provide strong inferences on the behavioural differences within these two important habitats of this one species.

### Tag data analysis

Analysis of tag data was performed using custom scripts in MATLAB 8.4 2014b (www.animaltags.org). Sensor data were decimated to a sampling rate of 25 Hz using identical symmetric finite impulse response low-pass filters on each channel. Accelerometer and magnetometer data were then calibrated and rotated from tag frame to whale frame to account for the orientation of the tag on the animal. Behavioural observations recorded during the focal follows on mother-calf pairs were used to inform classification of resting and active dives using the acceleration data of the tags. Minimum specific acceleration (MSA) was used as a proxy for movement effort (sensu)^20^. The instantaneous MSA, defined as the norm of the three accelerometer axes minus the acceleration due to gravitation of 1 g, was used to infer resting, foraging and “other” behaviour. Foraging dives were defined as having distinct jerk peaks generated by lunge feeding^20^. Resting periods were identified using epochs from the tag recordings that had concomitant data from detailed focal follows, allowing us to establish thresholds for resting versus active periods. Specifically, we did that by extracting MSA distribution for resting periods, this was done for each whale with sufficient focal follow data to verify epochs of resting. To exclude potential outliers in the MSA data we took the 95-percentile for these distributions. These outliers can derive from physical impacts on the accelerometer of the tag such as wave actions at the surface and physical contact between whales. We then used the mean value of these distributions as a threshold for resting to classify periods of resting during the rest of the tag outs. Specifically, a custom written script was used to evaluate mean MSA for every minute during the tag out to locate periods of resting, both submerged and at the surface. In total, we defined four behavioural categories: resting, foraging, suckling^21^, and other. The four categories were mutually exclusive: if an animal was not engaged in either resting or foraging (or in the case of calves, suckling), their behavior was categorized as “other”. Suckling was inferred through a combination of focal follow data and tag data (see Videsen *et al*.^21^ for a detailed description of how suckling behaviour was identified). Respiration rates were detected aurally from the DTAG recordings and were scored using a custom written auditing tool implemented in MATLAB. Student’s *t*-Tests were used to compare (a) respiration rates and (b) time spent within 3 m of the surface between foraging and breeding grounds. Assumptions of data normality and homogeneity of variance were checked and validated.

### Noise recordings and analysis

To quantify the soundscape during the peak season of mother-calf pair occurrence, four sound recorders (SoundTraps, www.oceaninstruments.co.nz, Fig. 1) were deployed along the western shore of Exmouth Gulf in 2014 in water depths of 7–14 meters. The sound recorders were deployed in areas with high densities of humpback whales^13^ but far enough apart (>10 km) that there was little, if any, overlap in the soundscape covered. They were moored at approximately 1.5 m above the seafloor between a 20 kg weight and a 200 mm subsurface buoy in the middle of the water column, and ran continuously for three days (sampling rate of 288 kHz, 16 bit, rendering a flat (±2 dB) frequency response from 0.02 to 100 kHz). Self-noise of recorders was measured in an anechoic room at the Danish Technical University, Lyngby, Denmark, while clip levels were established via the insert voltage calibration tones of each recorder (clip levels: 183–187 dB re 1 µPa). RMS noise levels were quantified in third octave bands in 1-minute blocks using custom written routines in MATLAB R2016a (Fig. 6) and evaluated as percentiles (5^th^, median and 95^th^) for a three-day period, where recorders were deployed at all four stations simultaneously. Three third-octave band frequencies (0.4, 1.25 and 10 kHz) with high levels of ambient noise (clear peaks in 95^th^ percentile) were further evaluated (Fig. 6, lower panels 1–4b). Two of these third-octave band frequencies, 0.4 and 1.25 kHz, showed clear events with higher levels of noise that exceeded the 95^th^ percentile (Fig. 6, lower panels 1–4b). These two frequency bands are both within humpback whale vocalizations^64,65^ and ship noise^66^. Thus to examine the sources giving rise to peaks in noise in four areas throughout the gulf, a threshold was set to the 95th percentile +6 dB for both frequency bands. Peaks above this threshold were found, and the maximum peak within each hour was evaluated by auditing a WAV file consisting of ±2 minutes relative to the peak time of noise using Adobe Audition 3.0, and was labelled according to the dominating noise source (Fig. 6, lower panels, 1–4b). To further evaluate the sources of noise in Exmouth Gulf, percentiles for a representative event of both a vessel passing and humpback whale sounds (Fig. 6,5a) were calculated, for comparison with overall percentiles to see their noise contribution. These percentiles were calculated for a 1-minute window around a peak event (Fig. 6, lower panels 2b, 4b and 5b,c) by using the same custom written routine as the main analysis, although with a 1-second analysis window to obtain third-octave levels and calculate percentiles (5^th^, median and 95^th^).

### Ethics statement and permit statement

The Australian field component and experimental protocols all were carried out and conformed to all relevant guidelines and regulations under permits issued by Murdoch University Animal Ethics Committee (R2594/13) and the Western Australian Department of Parks and Wildlife (DPaW; SF009641, CE004288). The Greenland field component and experimental protocols were all carried out and conformed to all relevant guidelines and regulations under permits issued by The Ministry of Fishing, Hunting and Agriculture, Greenland Self Government to the Greenland Institute of Natural Resources, according to §35 of the executive order no. 12 of December 22, 2014.

## Supplementary information

Supplementary Information

## References

[1] Stearns SC (1989). Trade-offs in life-history evolution. Brit. Ecol. Soc..

[2] Stephens PA, Boyd IL, McNamara JM, Houston AI (2009). Capital breeding and income breeding: their meaning, measurement, and worth. Ecology.

[3] Jönsson KI (1997). Capital and income breeding as alternative tactics of resource use in reproduction. Oikos.

[4] Dingle H, Drake A (2007). What is migration?. BioScience.

[5] Bengtson Nash SM (2018). Signals from the south; humpback whales carry messages of Antarctic sea‐ice ecosystem variability. Glob. Change Biol..

[6] Jenner KCS, Jenner MN, McCabe KA (2001). Geographical and temporal movements of humpback whales in Western Australian waters. APPEA Journ..

[7] Irvine LG, Thums M, Hanson CE, McMahon CR, Hindell MA (2017). Evidence for a widely expanded humpback whale calving range along the Western Australian coast. Mar. Mamm. Sci..

[8] Bannister J, Hedley SL (2001). Southern hemisphere group IV humpback whales: their status from recent aerial survey. Mem. Qld. Mus.

[9] Salgado Kent C, Jenner KCS, Jenner M, Bouchet P, Rexstad E (2012). Southern hemisphere breeding stock D humpback whale population estimates from North West Cape, Western Australia. J. Cetacean Res. Manage..

[10] IWC. *Report of the Scientific Committee Annex H – Other southern hemisphere whale stocks* (IWC, 2012).

[11] Hedley, S. L., Bannister, J. L. & Dunlop, R. A. Abundance estimates of southern hemisphere breeding stock ‘D’ humpback whales from aerial and land-based surveys off Shark Bay, Western Australia, 2008. *Cetacean Res*. *Manage*. Special Issue **3**, 209–21 (2011).

[12] Bejder M, Johnston D, Smith JN, Freidlaender A, Bejder L (2016). Embracing conservation success of recovering humpback whale populations: evaluating the case for downlisting the conservation status in Australia. Mar. Policy.

[13] Braithwaite JE, Meeuwig JJ, Jenner KCS (2012). Estimating cetacean carrying capacity based on spacing behaviour. Plos One.

[14] Chittleborough RG (1953). Aerial observations on the humpback whale, *Megaptera nodosa* (Bonnaterre), with notes on other species. Mar. Freshw. Res..

[15] Shire of Exmouth. *Multipurpose deep water wharf in Exmouth Gulf report* (Shire of Exmouth, 2015).

[16] NRC. *Ocean Noise and Marine Mammals* (National Academy Press, 2003). 25057640

[17] Hildebrand JA (2009). Anthropogenic and natural sources of ambient noise in the ocean. Mar. Ecol. Prog. Ser..

[18] Tyack PL (2008). Implications for marine mammals of largescale changes in the marine acoustic environment. J. Mammal..

[19] Richardson, W. J., Greene, C. R. J., Malme, C. I. & Thomson, D. H. *Marine Mammals and Noise* (Academic Press, 1995).

[20] Simon M, Johnson M, Madsen PT (2012). Keeping momentum with a mouthful of water: behavior and kinematics of humpback whale lunge feeding. J. Exp. Biol..

[21] Videsen S, Bejder L, Johnston M, Madsen P (2017). High suckling rates and acoustic crypsis of humpback whale neonates maximize potential for mother-calf energy transfer. Funct. Ecol..

[22] Christiansen F, Dujon AM, Sprogis KR, Arnould JPY, Bejder L (2016). Non-invasive unmanned aerial vehicle provides estimates of the energetic cost of reproduction in humpback whales. Ecosphere.

[23] UNCTAD. *Review of Maritime Transport* (UNCTAD, 2016).

[24] Magera AM, Flemming JE, Kaschner K, Christensen LB, Lotze HK (2013). Recovery trends in marine mammal populations. Plos One.

[25] Rockwood RC, Calambokidis J, Jahncke J, Li S (2017). High mortality of blue, humpback and fin whales from modeling of vessel collisions on the U.S. West Coast suggests population impacts and insufficient protection. Plos One.

[26] Constantine, R. *et al*. Mitigation of vessel-strike mortality of endangered Bryde’s whales in the Hauraki Gulf, New Zealand. *Biol*. *Conserv*. **186**, 149–157 (2015).

[27] IWC. Ship strike summary data, https://iwc.int/index.php?cID=872&cType=document (2010).

[28] Cates, K. *et al*. *Strategic plan to mitigate the impacts of ship strikes on cetacean populations: 2017*–*2020* (IWC, 2017).

[29] Peel D, Smith JN, Childerhouse S (2018). Vessel strike of whales in Australia: The challenges of analysis of historical incident data. Fr. Mar. Sci..

[30] Nowacek DP, Johnson MP, Tyack PL (2004). North Atlantic right whales (*Eubalaena glacialis*) ignore ships but respond to alerting stimuli. Proc R Soc Lond B Biol Sci..

[31] Kraus SD (2005). North Atlantic right whales in crisis. Science..

[32] Forrest TG, Miller GL, Zagar JR (1993). Sound propagation in shallow water: implications for acoustic communication by aquatic animals. Bioacoustics.

[33] Knudsen VO, Alford RS, Emling JW (1948). Underwater ambient noise. J. Mar. Res..

[34] Cato, D. H. & Bell, M. J. Ultrasonic ambient noise in Australian shallow water at frequencies up to 200 kHz (*Materials research labs*, 1992).

[35] Møhl, B. Masking effects of noise; their distribution in time and space. In *The question of sound from icebreaker operations*, *the proceedings of a workshop* (ed. Peterson, N. M.) 259–266 (Arctic Pilot Project, 1981).

[36] Brenowitz EA (1982). The active space of red-winged blackbird song. J. Comp. Physiol..

[37] Jensen FH (2009). Vessel noise effects on delphinid communication. Mar. Ecol. Prog. Ser..

[38] Herman LM (2017). The multiple functions of male song within the humpback whale (*Megaptera novaeangliae*) mating system: review, evaluation, and synthesis. Biol. Rev..

[39] Pitman RL (2015). Whale killers: prevalence and ecological implications of killer whale predation on humpback whale calves off Western Australia. Mar. Mamm. Sci..

[40] Hermannsen L, Beedholm K, Tougaard J, Madsen PT (2014). High frequency components of ship noise in shallow water with a discussion of implications for harbor porpoises (*Phocoena phocoena*). J. Acoust. Soc. Am..

[41] Baker, C. S. & Herman, L. M. *Behavioral responses of summering humpback whales to vessel traffic: experimental and opportunistic observations*. Final report to the National Park Service, Alaska Regional Office (United States Department of the Interior, 1989).

[42] Bauer GB, Mobley JR, Herman L (1993). Responses of wintering humpback whales to vessel traffic. J. Acoust. Soc. Am..

[43] Blair HB, Merchant ND, Friedlaender AS, Wiley DN, Parks SE (2016). Evidence for ship noise impacts on humpback whale foraging behaviour. Biol. Letters..

[44] Miller PJO, Biassoni N, Samuels A, Tyack PL (2000). Whale songs lengthen in response to sonar. Nature.

[45] Sousa-Lima RS, Clark CW (2008). Modeling the effect of boat traffic on the fluctuation of humpback whale singing activity in the Abrolhos National Marine Park, Brazil. Can. Acoust..

[46] Scheidat M, Castro C, Gonzalez J, Williams R (2004). Behavioural responses of humpback whales (*Megaptera novaeanglia*e) to whale watching boats near Isla de la Plata, Machalilla National Park, Ecuador. J. Cetacean Res. Manage..

[47] Nowacek DP, Thorne LH, Johnston DW, Tyack PL (2007). Responses of cetaceans to anthropogenic noise. Mammal Rev..

[48] Dunlop RA, Cato DH, Noad MJ (2008). Non-song acoustic communication in migrating humpback whales (*Megaptera novaeangliae*). Mar. Mamm. Sci..

[49] Morete ME, Bisi TL, Rosso S (2007). Mother and calf humpback whale responses to vessels around the Abrolhos Archipelago, Bahia, Brazil. J. Cetac. Res. Manage..

[50] Sprogis, K. R., Bejder, L. & Christiansen, F. *Swim-with-whale tourism trial in the Ningaloo Marine Park*, *Western Australia*. Report to the Department of Parks and Wildlife (Murdoch University 2017).

[51] Silbert GK, Adams JD, Bettridge S (2012). Vessel operator response to a voluntary vessel/whale collision reduction measure. Endanger. Species Res..

[52] Van der Hoop J, Vanderlaan ASM, Taggart CT (2012). Absolute probability of lethal vessel strikes to North Atlantic right whales in Roseway Basin, Scotian Shelf. Ecol. Appl..

[53] NOAA (2008). Endangered fish and wildlife; final rule to implement speed restrictions to reduce the threat of ship collisions with North Atlantic right whales. Fed. Regist..

[54] Conn PB, Silber GK (2013). Vessel speed restrictions reduce risk of collision related mortality for North Atlantic right whales. Ecosphere.

[55] Van der Hoop J (2014). Vessel strikes to large whales before and after the 2008 ship strike rule. Conserv. Lett..

[56] Gende S (2011). A Bayesian approach for understanding the role of ship speed in whale-ship encounters. Ecol. Appl..

[57] Campbell-Malone R (2008). Gross and histologic evidence of sharp and blunt trauma in North Atlantic right whales (*Eubalaena glacialis*) killed by vessels. J. Zoo Wildl. Med..

[58] Arveson P, Vendittis DJ (2000). Radiated noise characteristics of a modern cargo ship. J. Acoust. Soc. Am..

[59] Ross, D. Cavitation. In *Mechanics of Underwater Noise* (Pergamon Press, 1976).

[60] Johnson MP, Tyack PL (2003). A digital acoustic recording tag for measuring the response of wild marine mammals to sound. IEEE J. Oceanic Eng..

[61] Boye T, Simon M, Madsen PT (2010). Habitat use of humpback whales in Godthaabsfjord, West Greenland, with implications for commercial exploitation. J. Mar. Biol. Assoc. UK.

[62] Stevick PT (2003). Segregation of migration by feeding ground origin in North Atlantic humpback whales (*Megaptera novaeangliae*). J. Zool. London..

[63] Moore MJ (2001). Ultrasonic measurement of blubber thickness in right whales. J. Cetac. Res. Manage..

[64] Au WWL (2006). The acoustic properties of humpback whale songs. J. Acoust. Soc. Am..

[65] Stimpert AK, Au WWL, Parks SE, Hurst T, Wiley DN (2011). Common humpback whale (*Megaptera novaeangliae*) sound types for passive acoustic monitoring. J. Acoust. Soc. Am..

[66] Wenz GM (1962). Acoustic ambient noise in the ocean: spectra and sources. J. Acoust. Soc. Am..

