# Supplementary material for: Low energy expenditure and resting behaviour of humpback whale mother-calf pairs highlights conservation importance of sheltered breeding areas
Source: Sci Rep. 2019 Jan 25;9:771. doi: 10.1038/s41598-018-36870-7 (PMC6347592; doi:10.1038/s41598-018-36870-7)
Supplement: Supplementary file 1 — Supplementary Information [file 41598_2018_36870_MOESM1_ESM.pdf]

SUPPLEMENTARY MATERIAL

Low energy expenditure and resting behaviour of humpback whale mother-calf pairs highlights conservation importance of sheltered breeding areas

Bejder, L, Videsen, S, Hermannsen, L, Simon, M., Hanf, D. and Madsen, P.T.

Table S1. Overview of the 25 tagged humpback whales in Exmouth (EX), Western Australia and 16 in Greenland (Godthaabsfjord, GF; Disko Bay, DB). LF: Lactating female; N: Neonate; AM: adult male; Adult: A.

Date (dd.mm.year)	General location	Tag name	Lat	Lon	Age/sex category	Tag on time (hrs)
11.09.2013	EX	mn13_254a	S 22.310	E 114.256	LF	21
11.09.2013	EX	mn13_254b	S 22.310	E 114.256	LF	2.5
12.09.2013	EX	mn13_255a	S 22.277	E 114.244	LF	22
16.09.2013	EX	mn13_259a	S 22.276	E 114.226	LF	19
16.09.2013	EX	mn13_259b	S 22.268	E 114.227	LF	7
16.09.2013	EX	mn13_259c	S 22.168	E 114.135	LF	20
18.09.2013	EX	mn13_261a	S 22.319	E 114.211	LF	24
19.09.2013	EX	mn13_262a	S 22.328	E 114.221	LF	1.7
19.09.2013	EX	mn13_262b	S 22.285	E 114.194	LF	24
20.09.2013	EX	mn13_263a	S 22.308	E 114.203	LF	4
21.09.2013	EX	mn13_264a	S 22.358	E 114.261	LF	24
22.09.2013	EX	mn13_265a	S 22.297	E 114.191	LF	24
23.09.2013	EX	mn13_266a	S 22.307	E 114.198	LF	15.5
24.09.2013	EX	mn13_267a	S 22.328	E 114.198	LF	9
26.08.2014	EX	mn14_238a	S 22.270	E 114.188	N	6
26.08.2014	EX	mn14_238b	S 22.254	E 114.185	LF	3
27.08.2014	EX	mn14_239a	S 22.144	E 114.143	N	4
28.08.2014	EX	mn14_239b	S 22.151	E 114.142	LF	3.5
29.08.2014	EX	mn14_241a	S 22.371	E 114.226	N	3
30.08.2014	EX	mn14_242a	S 22.307	E 114.257	N	6.25
31.08.2014	EX	mn14_243a	S 22.341	E 114.264	N	7.5
03.09.2014	EX	mn14_246b	S 22.308	E 114.240	N	24
04.09.2014	EX	mn14_247a	S 22.197	E 114.250	N	8.75
04.09.2014	EX	mn14_247b	S 22.223	E 114.258	N	6.25
03.09.2014	EX	mn14_246a	S 22.357	E 114.228	AM	8.25
29.06.2007	GF	mn07_180a	N 64.101	W 51.459	A	6.05
11.07.2007	GF	mn07_192a	N 64.139	W 51.465	A	4.88
22.07.2007	GF	mn07_203a	N 64.18	W 51.76	A	25.28
25.05.2008	GF	mn08_146a	N 69.230	W 53.768	A	5.83
31.05.2008	GF	mn08_152a	N 69.230	W 53.768	A	4.87
01.06.2008	GF	mn08_153a	N 64.223	W 51.566	A	7.51
03.06.2008	GF	mn08_155a	N 64.229	W 51.251	A	4.76
04.06.2008	GF	mn08_156a	N 64.223	W 51.379	A	5.84
06.06.2008	GF	mn08_158a	N 64.137	W 51.079	A	3.57
08.06.2008	GF	mn08_160a	N 64.127	W 51.222	A	5.26
11.06.2008	GF	mn08_163a	missed	missed	A	2.37

26.06.2012	DB	mn12_178a	N 68.74	W 52.55	A	7.42
26.06.2012	DB	mn12_178b	N 68.74	W 52.55	A	5.96
27.06.2012	DB	mn12_179a	N 68.74	W 52.55	A	6.37
28.06.2012	DB	mn12_180a	N 68.74	W 52.55	A	6.45
02.07.2012	DB	mn12_184a	N 68.74	W 52.55	A	7.23
03.07.2013	DB	mn12_185a	N 68.74	W 52.55	A	11.13

Figure S1: Dive profiles (blue; depth in meters) and MSA (red; m/s^2) of adult whales on a breeding ground (Exmouth; n=17; 16 lactating females and one adult male (mn246a). Grey shading indicates time from sunset to sunrise. The proportion of time each whale spent at various depths is depicted on the right-hand side of the plot.

Figure S2: Dive profiles (blue; depth in meters) and MSA (red; m/s2) of adult whales on a foraging ground (Greenland; n=16). Grey shading indicates time from sunset to sunrise (for the whales tagged in 2012 the sun was up 24h a day). The proportion of time each whale spent at various depths is depicted on the right-hand side of the plot.

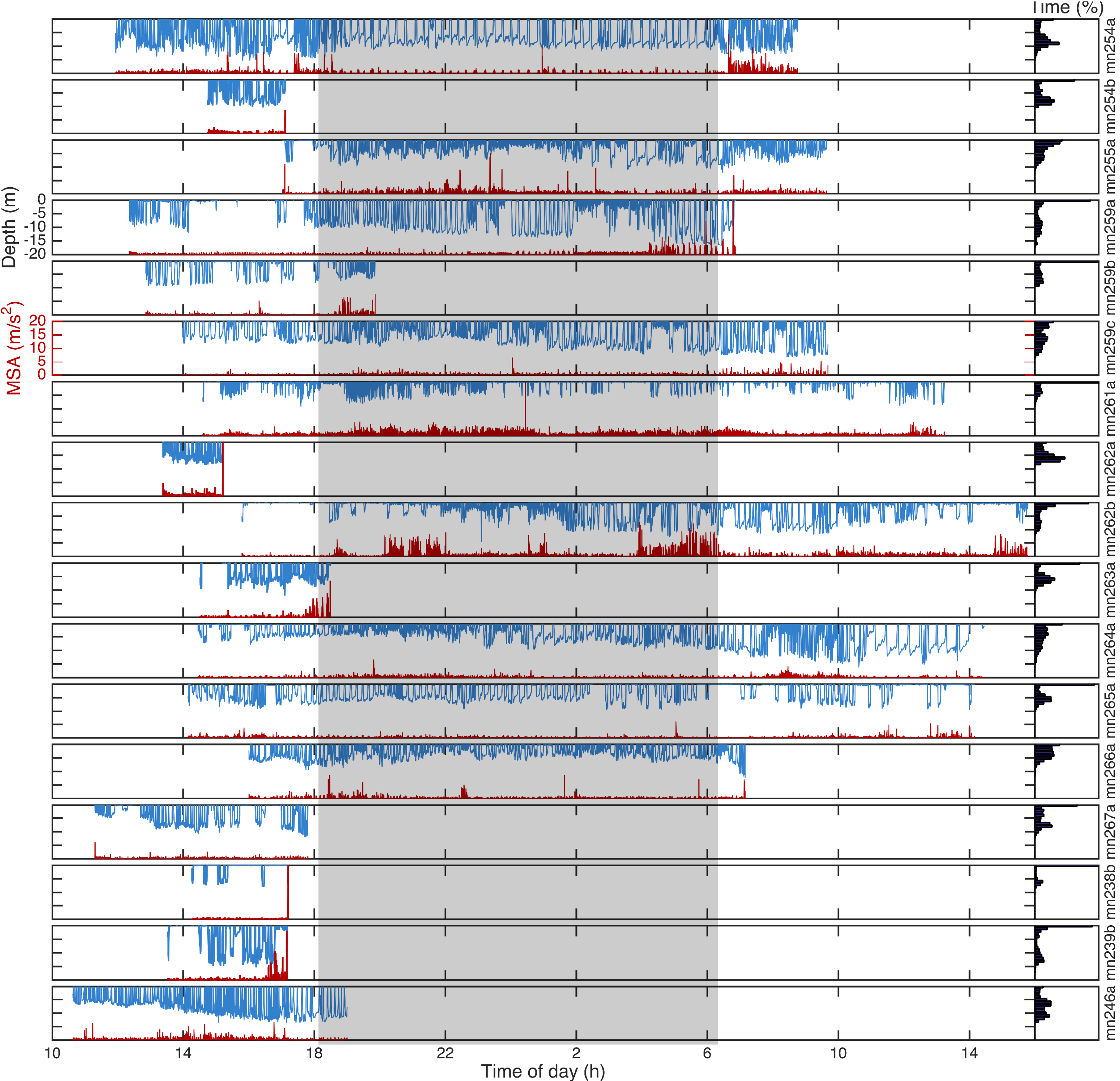

Time (%)
Depth (m)
0
-5
-10
-15
-20
MSA (m/s^2)
20
15
10
5
0
mn254a
mn254b
mn255a
mn259a
mn259b
mn259c
mn261a
mn262a
mn262b
mn263a
mn264a
mn265a
mn266a
mn267a
mn238b
mn239b
mn246a
10
14
18
22
2
6
10
14
Time of day (h)

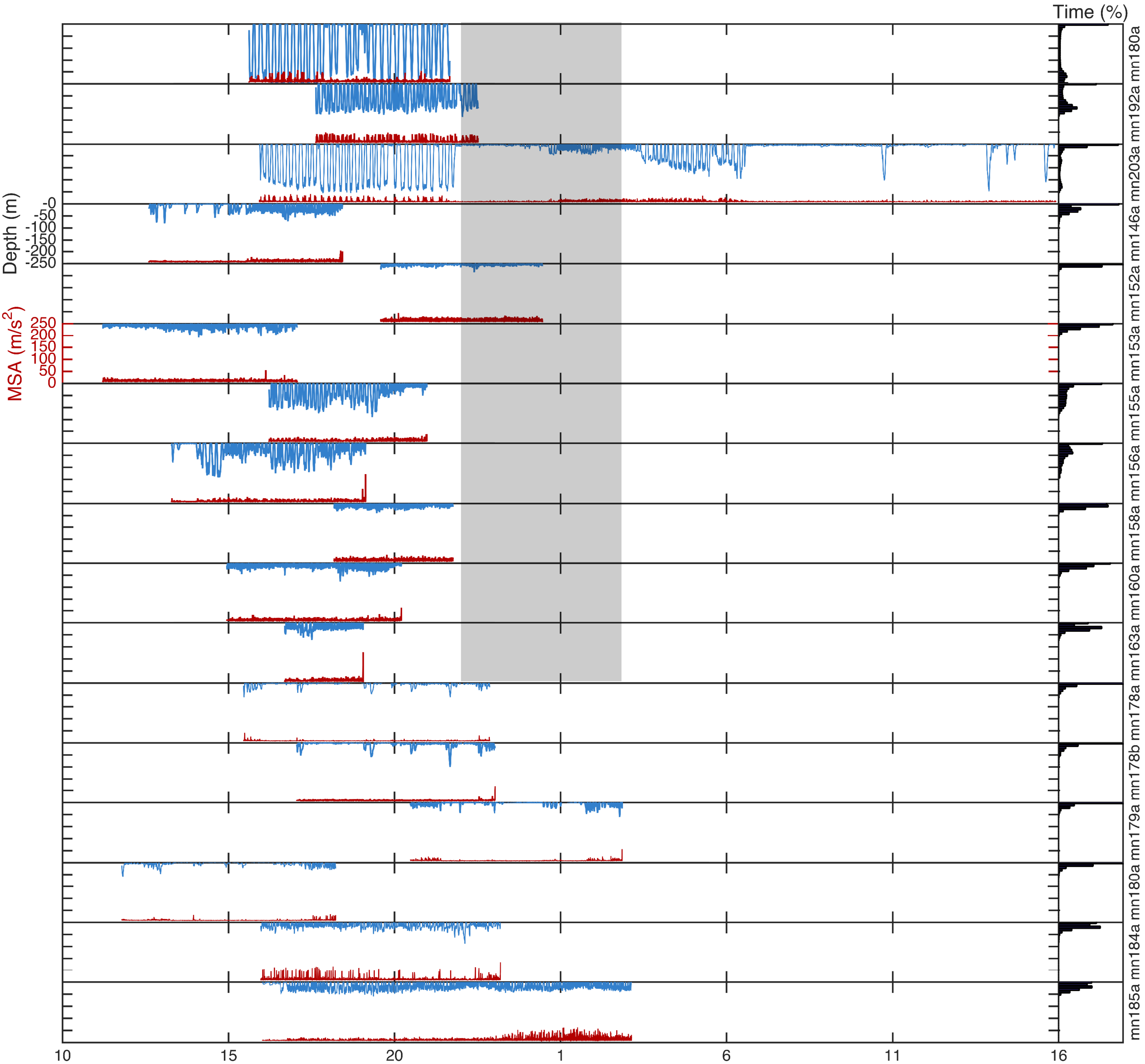

Time (%)
Depth (m)
-0
-50
-100
-150
-200
-250
MSA (m/s^2)
250
200
150
100
50
0
mn180a
mn192a
mn203a
mn146a
mn152a
mn153a
mn155a
mn156a
mn158a
mn160a
mn163a
mn178a
mn178b
mn179a
mn180a
mn184a
mn185a
10
15
20
1
6
11
16
